# Adaptive Kalman Filter Fusion Positioning Based on Wi-Fi and Vision

**DOI:** 10.3390/s25030671

**Published:** 2025-01-23

**Authors:** Shuxin Zhong, Li Cheng, Haiwen Yuan, Xuan Li

**Affiliations:** College of Electrical Information, Wuhan Institute of Technology, Wuhan 430205, China; 22303010011@stu.wit.edu.cn (S.Z.); hw_yuan@wit.edu.cn (H.Y.); lixuan@wit.edu.cn (X.L.)

**Keywords:** indoor positioning, adaptive Kalman filtering, Wi-Fi positioning, visual positioning

## Abstract

The fusion of multiple sensor data to improve positioning accuracy and robustness is an important research direction in indoor positioning systems. In this paper, a Wi-Fi- and vision-based Fusion Adaptive Kalman Filter (FAKF) method is proposed for improving the accuracy of indoor positioning. To improve the accuracy of Wi-Fi positioning, a random forest algorithm with added region restriction is proposed. For visual positioning, YOLOv7 target detection and Deep SORT target tracking algorithms are combined in order to improve the stability of visual positioning. The fusion positioning method proposed in this study uses Kalman filtering for state estimation and updating by combining measurements from camera and Wi-Fi sensors, and it adaptively adjusts the parameters and weights of the filters by monitoring the residuals of the camera and Wi-Fi measurements in real time in order to optimize the accuracy and stability of the position estimation. In the experimental section, the real trajectory data and the predicted trajectory data generated using different positioning methods are compared. The experimental results show that the fused positioning method can significantly reduce positioning errors and the fused data can more accurately reflect the actual position of a target compared with single-sensor data.

## 1. Introduction

In the current era of information technology, accurate indoor positioning technology has become a key technology in the fields of intelligent navigation, environmental monitoring, emergency rescue and personal services. Although the Global Positioning System (GPS) [1] provides high-precision positioning services in outdoor environments, its positioning accuracy is greatly reduced in indoor environments due to signal attenuation and multipath effects, so outdoor positioning and navigation technology cannot be directly applied to indoor environments and indoor positioning technology has become indispensable as Internet of Things (IoT) technology has developed and become popular [2]. How to improve the accuracy of indoor positioning has become more and more urgent, and positioning has become a research hotspot in academia and the information technology industry. In recent years, Wi-Fi-based positioning technology and vision-based positioning technology have been widely considered for their unique advantages [3].

Wi-Fi positioning technology mainly utilizes the signal strength emitted by deployed wireless access points (APs) in indoor environments to estimate the position of the user [4]. The advantages of this technology are its low deployment cost, wide coverage, and no additional hardware support. However, the main challenge of Wi-Fi positioning is that its positioning accuracy is greatly affected by environmental interference, such as human movement, multipath propagation, indoor temperature changes and other factors that lead to changes in signal strength, thus affecting positioning accuracy.

On the other hand, visual positioning techniques determine a location by analyzing the image information captured by a camera, and they usually rely on image recognition and machine learning techniques to achieve positioning by identifying feature points in an image [5]. The accuracy of visual positioning is high in a small area, but in indoor environments with a large field of view, the positioning accuracy significantly decreases. This is because it may be difficult to accurately match the feature points in an image over a large range, leading to an increase in positioning deviation. Also, visual positioning requires processing a large amount of image data during processing, which has a high demand on computational resources and performs erratically in environments with poor lighting conditions or restricted fields of view.

In order to overcome the limitations of a single technique, the method of fusing multiple sensor data for positioning has gradually become a research hotspot [6]. Kalman filtering is an effective data fusion technique widely used in signal processing and control systems [7]. It achieves the optimal estimation of a system state by establishing a dynamic model of the system and continuously updating and predicting the system state using the observed data. However, although Kalman filtering performs well in fusing different types of sensor data, its application in complex indoor environments remains challenging. Current research has mostly focused on the use of Kalman filtering for fusing a few types of sensor data, such as inertial navigation (IMU) and GPS, with less attention to combining Wi-Fi signals with visual information for positioning [8].

Therefore, this paper proposes a Kalman filter positioning technique that fuses Wi-Fi signals and visual information. Wi-Fi signal positioning has a low accuracy in the case of occlusion and multipath effects, while visual information positioning relies on complex image processing algorithms. In complex environments with a large field of view, the accuracy of visual positioning significantly decreases and is computationally intensive and significantly affected by ambient lighting. The effective fusion of data from two sensors through Kalman filtering can compensate for their respective deficiencies to a certain extent, thus improving the accuracy and robustness of indoor positioning. This method can not only improve the positioning accuracy but also provide a new solution to the positioning problem in complex indoor environments. Therefore, the research in this paper has important theoretical and practical significance. The main contributions of this study are as follows:

1. In the implementation of the Wi-Fi positioning component, a random forest algorithm incorporating area restriction is proposed in this study to enhance the accuracy of Wi-Fi positioning in terms of its adaptability to environmental changes.
2. In the development of the visual positioning component, YOLOv7 target detection and Deep SORT target tracking algorithms are introduced to optimize the efficiency and accuracy of image processing and effectively improve the stability of visual positioning.
3. An adaptive Kalman filter positioning technique fusing Wi-Fi signals and visual information is proposed. The technique uses Wi-Fi signal strength and visual information for preliminary positioning analysis, and it dynamically adjusts the parameters and weights of the filter by monitoring the residuals of the camera and Wi-Fi measurements in real time. The technique effectively integrates the two types of sensing data and aims to improve the accuracy and stability of the indoor positioning system.
4. Through experimental verification in a real environment, this study could significantly reduce the uncertainty of each independent technique and show good positioning performance in complex indoor environments so as to achieve more accurate and robust positioning effect, which has a wide range of application prospects and practical value.

## 2. Related Work

In the past few decades, indoor positioning through technologies such as Wi-Fi [9], Bluetooth [10], Radio-Frequency Identification (RFID) [11], Ultra-Wideband (UWB) [12], cameras [13], and sensors in wearable [14] and smartphone devices [15] has been a significant area of research aimed at addressing limitations related to positioning accuracy, coverage area, and complexity. This paper provides an overview of the relevant research findings from both wireless positioning and visual positioning perspectives.

Wi-Fi-based indoor positioning methods include triangulation [16], fingerprint matching [17], time difference [18], angle, and machine-learning-based methods [19]. Among these, RSSI-based fingerprint positioning methods dominate the field, with their accuracy significantly influenced by the density and calibration levels of signal collection. Hemin Ye and Jiansheng Peng enhanced traditional Wi-Fi fingerprinting algorithms by increasing the density and quality of data acquisition, standardizing the processing of Wi-Fi signal strength values, adopting a similarity matching method based on the Mahalanobis distance, and integrating an improved adaptive k-value WKNN algorithm. These improvements significantly enhanced the accuracy of indoor Wi-Fi localization [20]. Additionally, Ghazaleh K et al. integrated Ultra-Wideband (UWB) and Wi-Fi technologies. They proposed an algorithm that combines Received Signal Strength (RSS) values with Gaussian Process (GP) fusion for distance measurement, utilizing UWB Two-Way Ranging (TWR) and Wi-Fi Round Trip Time (RTT) to calculate distance values [21].

With the development of deep learning, the advantages of visual positioning have become increasingly apparent. Shuang L et al. proposed a method for classifying different scenes based on Deep Belief Networks, which can obtain positioning information from photographs of a surrounding environment [22]. Jingjing Y et al. introduced a novel three-dimensional (3D) passive visually-assisted Pedestrian Dead Reckoning (PDR) system utilizing multiple surveillance cameras and PDR. By integrating inertial navigation and a Faster Region Convolutional Neural Network (Faster R-CNN), this system continuously tracks user movement across different floors [23]. Wen L et al. developed a low-rank fusion-based positioning model for Wi-Fi and visual fingerprinting (LRF-WiVi) that leverages the complementarity of heterogeneous signals through the end-to-end interactive modeling of signal-specific actions and positioning information [24]. Ying C et al. proposed a two-stage indoor positioning system combining Wi-Fi fingerprinting, visual surveillance cameras, and PDR (iWVP). This system initially employs Wi-Fi fingerprinting for rough localization and subsequently integrates data from surveillance cameras and IMU sensors for precise positioning [25]. Tang et al. proposed a visually guided fingerprint positioning (KV) technique based on Wi-Fi coarse positioning and fusion positioning in two stages. The positioning accuracy and stability were improved by a two-metric adaptive KNN method and multi-angle unsupervised fusion positioning [26].

However, the accuracy of indoor positioning based on Wi-Fi is greatly affected by environmental interference. On the other hand, visual positioning techniques, which rely on the recognition of feature points in image information, significantly decrease in accuracy in complex environments with a large field of view. Therefore, this paper proposes an adaptive Kalman filter fusion positioning method based on Wi-Fi and vision. Experiments were conducted in real-world scenarios (complex indoor environments with occlusions) to validate and test the positioning method across different paths.

## 3. System Overview

**Figure 1:**
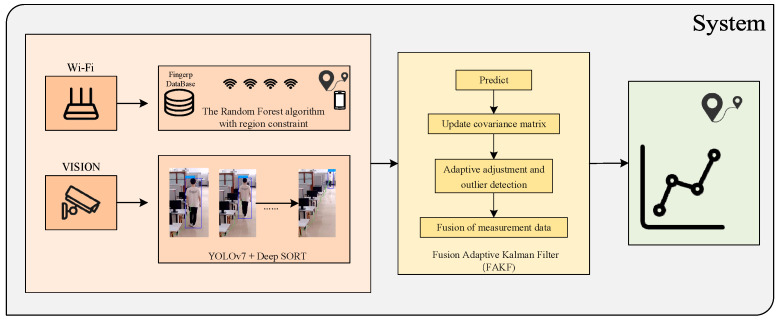
System framework.

The framework diagram of this system is shown in Figure 1. It consists of three main core modules: the Wi-Fi positioning module, the visual positioning module and the adaptive Kalman filter (FAKF) data fusion module.

In the Wi-Fi positioning module, the system processes Wi-Fi signal strength data from multiple points using a random forest algorithm with added area restrictions. This approach enhances the system’s ability to adapt to changes in the environment and provides more accurate location estimates. The visual positioning module processes image data collected by the camera for target identification and tracking by integrating the YOLOv7 object detection algorithm and the Deep SORT object tracking algorithm. The YOLOv7 algorithm excels in target detection with high efficiency and accuracy, while the Deep SORT algorithm further improves the stability and continuity of target tracking. Combining the two effectively improves the stability and continuity of pedestrian positioning estimation. The FAKF data fusion module fuses Wi-Fi positioning and visual positioning through adaptive Kalman filtering. The module consists of five main steps: prediction, covariance matrix updating, adaptive tuning, outlier detection, and measurement data fusion. By utilizing the adaptive Kalman filter, the system effectively reduces the uncertainty associated with each positioning technique, thus providing more accurate positioning results.

## 4. Proposed Methodology

### 4.1. Wi-Fi-Based Indoor Positioning

This study introduces a machine-learning-based Wi-Fi indoor positioning method utilizing a random forest algorithm enhanced by regional constraints for location prediction. This methodology significantly augments the accuracy and efficiency of indoor positioning.

Wi-Fi data are collected to establish an offline fingerprint database. During the positioning phase, the system processes online Received Signal Strength (RSS) data for location prediction. In the initialization phase, the prediction point is set as the coordinate origin (0,0), with an offset defined as a constraint on the search area. To mitigate initial errors, the system disregards the first online RSS data point, processing subsequent RSS data sequentially. For each data point, the algorithm identifies RSS values and corresponding position data from the offline dataset that are within the offset of the current prediction point. These data points constitute the training dataset for that time step. The online RSS data are fed into a pre-trained regression model, allowing the algorithm to predict the current location. Each prediction result is then used as the initial point for the next time step, ensuring continuity and stability in predictions. Moreover, to maintain the real-time accuracy of the training dataset, the algorithm updates the RSS and position information within the restricted area after each prediction.

The random forest algorithm with added region restriction proposed in this study employs several measures in the computation of node impurity, including mean square error (*MSE*), mean absolute error (${MAE}_{single\;dimension}$), and Poisson deviation (*Poisson*):

(1) $$MSE=\frac{1}{N}\sum_{i=1}^{N}{\left(y_{i}-{\hat{y}}_{i}\right)}^{2}$$

(2) $${MAE}_{single\;dimension}=\frac{1}{N}\sum_{i=1}^{N}\left|y_{i}-{\hat{y}}_{i}\right|$$

(3) $$Poisson=\sum_{i=1}^{N}\left(y_{i}\log\left(\frac{y_{i}}{{\hat{y}}_{i}}\right)-\left(y_{i}-{\hat{y}}_{i}\right)\right)$$

where $y_{i}$ is the observed count data, ${\hat{y}}_{i}$ is the count data predicted by the model, and *N* is the total number of samples.

The impurity reduction of nodes is calculated as in Equation (4):

(4) $$∆Imy=\frac{N_{t}}{N}\left(Imy-\frac{N_{tL}}{N_{t}}{Imy}_{L}-\frac{N_{tR}}{N_{t}}{Imy}_{R}\right)$$

where *N* is the total number of samples, $N_{t}$ is the number of current node samples, and $N_{tL}$ and $N_{tR}$ are the number of samples of the left and right nodes, respectively.

The positioning accuracy is evaluated by measuring the Euclidean distance between the predicted and actual positions, thus verifying the effectiveness of the proposed method. The Wi-Fi indoor positioning algorithm proposed in this study can quickly adapt to environmental changes by dynamically adjusting its training dataset. Depending on the input parameters, the algorithm can select either K-Nearest Neighbors (KNN) or random forest regression models, which improves the adaptability and flexibility of the system. Highly accurate indoor positioning in complex environments can be achieved by accurately integrating online and offline RSS data and by selecting appropriate training data in restricted areas.

### 4.2. Vision-Based Indoor Positioning

#### 4.2.1. Pedestrian Detection and Tracking Based on Video Images

Pedestrian detection and pedestrian tracking are core components of vision-based indoor positioning systems. In this study, a YOLOv7 target detection framework was used to detect pedestrians in video clips and combined with the Deep SORT algorithm to achieve pedestrian tracking. The main reason for choosing YOLOv7 is that it achieves a more balanced performance in terms of accuracy, inference speed and model optimization. Compared with YOLOv5, YOLOv7 makes significant improvements in model architecture and training strategy, such as the introduction of an adaptive anchor frame mechanism and optimized loss function design, which improve the accuracy of target detection in complex scenes. Additionally, YOLOv7’s improvement in small target detection capability is particularly significant, which is important for pedestrian detection in indoor environments with complex backgrounds.

Although YOLOv8 exhibits a higher detection accuracy and more advanced feature extraction capabilities in certain tasks, its training and inference process is more demanding on computational resources and is not as efficient as YOLOv7 under conditions of limited hardware resources. Therefore, combining the dual considerations of detection accuracy and resource efficiency, YOLOv7 was finally selected in this study to achieve efficient and reliable pedestrian detection and tracking.

YOLOv7 employs a single-stage detection strategy that divides an input image into grids and predicts target presence, bounding boxes, and category labels for each grid cell. Its model architecture integrates state-of-the-art feature extraction networks (e.g., DarkNet, ResNet, or EfficientNet) that are pre-trained to efficiently capture complex image features. In addition, YOLOv7 incorporates feature fusion techniques with Feature Pyramid Networks (FPNs) to achieve robust target detection across scales, which is crucial in pedestrian recognition at different viewing angles and distances.

In terms of loss function design, YOLOv7 employs a combination of localization loss, classification loss and target confidence loss to improve the accuracy of target location and category prediction [27]. A non-maximum suppression (NMS) algorithm is applied in the detection post-processing stage, which eliminates redundant bounding boxes by calculating the intersection and integration ratio (*IoU*) of the bounding boxes to ensure the accuracy of the final detection results, as shown in Equation (5). The above optimization strategy further enhances the target detection and tracking capability of YOLOv7 in complex indoor environments, making it an ideal choice for this study.

(5) $$IoU=\frac{Detection\;Result\;\cap \;Detection\;Truth}{Detection\;Result\;\cup \;Detection\;Truth}$$

Deep SORT, standing for Deep Association Metric Learning for Tracking, integrates a deep learning model that learns a target’s epigenetic features to improve tracking accuracy in dynamic scenarios [28]. It employs Kalman filtering to predict the target’s state in subsequent frames, while the Hungarian algorithm performs data association using the predicted state and observed data, followed by state updates based on the association results.

Combining YOLOv7 with Deep SORT, this study effectively enhances the accuracy of pedestrian detection and tracking in complex environments, thus bolstering support for vision-based indoor positioning systems.

#### 4.2.2. Coordinate Transformation Models

Monocular cameras are widely used in most indoor surveillance environments. However, monocular cameras have difficulties in acquiring target depth information, and their acquired target pixel coordinates do not directly correspond to the actual spatial position of a target. Therefore, this study aimed to achieve a conversion from the pixel coordinates acquired by monocular cameras into world coordinates (describing the actual spatial position of a target). Based on the camera imaging principle, a checkerboard-based camera calibration method [29] was used to achieve the “pixel-to-world” coordinate conversion.

The specific process of coordinate conversion consists of the following two steps: firstly, the points in the world coordinate system are converted into the camera coordinate system using an external reference matrix; secondly, the points in the camera coordinate system are projected onto the imaging plane using an internal reference matrix to obtain the pixel coordinates. Equation (6) describes how the points $\left(x_{w},y_{w},z_{w}\right)$ in the 3D world coordinate system are converted into pixel coordinates (*u*, *v*) on the imaging plane using the internal and external references of the camera.

(6) $$Z_{c}\left[\begin{array}{c} u\\ v\\ 1 \end{array}\right]=\left[\begin{array}{ccc} f\times \frac{1}{d_{x}} & 0 & u_{0}\\ 0 & f\times \frac{1}{d_{y}} & v_{0}\\ 0 & 0 & 1 \end{array}\right]\left[\begin{array}{cc} R & T \end{array}\right]\left[\begin{array}{c} x_{w}\\ y_{w}\\ z_{w}\\ 1 \end{array}\right]$$

**Figure 2:**
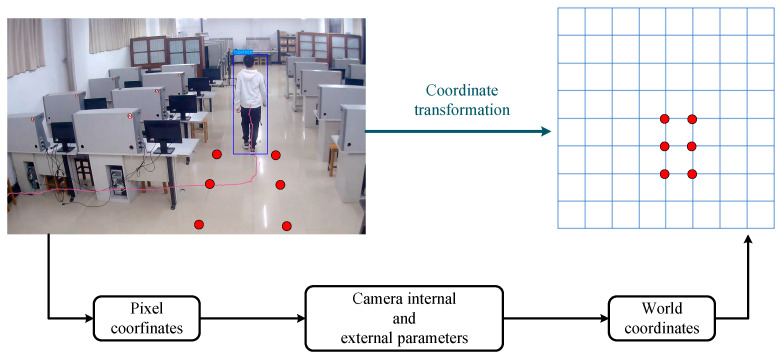
Architecture of the coordinate transformation model.

Among other things, the internal reference matrix (Intrinsic Matrix) contains the focal length *f*, the pixel dimensions $d_{x}$ and $d_{y}$ (the number of pixels per unit length), and the photocentric coordinates $u_{0}$ and $v_{0}$ of the imaging coordinate system (which indicate the position of the centre point of the imaging plane in the pixel coordinate system). The internal reference matrix reflects the properties of the camera itself, and different cameras have different internal reference matrices. The external reference matrix contains the rotation matrix *R*, which describes the rotation transformation from the world coordinate system to the camera coordinate system, and the translation vector *T,* which describes the translation transformation from the world coordinate system to the camera coordinate system. The parameters in the equations can be obtained by camera calibration. The architecture of the coordinate transformation model is shown in Figure 2.

Through the above steps, the conversion from pixel coordinates into world coordinates can be successfully achieved, providing theoretical support for indoor positioning based on monocular cameras.

### 4.3. Fusion Positioning Based on Adaptive Kalman Filtering

This study presents an improved adaptive Kalman filtering (FAKF) algorithm, a data-layer fusion method designed to significantly reduce the localization errors caused by sensor drift. The algorithm employs an observability-based data fusion strategy to enhance localization accuracy. Specifically, it establishes a potential correspondence between wireless signal strength and visual signals for each target by integrating the receiver’s wireless signal strength (W-data) with the positional coordinates of the person in the video frame (V-data).

The FAKF algorithm comprises four main components: information reset, time update, measurement update, and information fusion between wireless and visual signals. The algorithm represents the position of the WV data through the state error, facilitating the effective fusion of W and V data.

At each time step, the Kalman filter performs a prediction step to estimate the state and covariance for the next time step, as described by the following equations:

(7) $${\hat{x}}_{t}^{-}=F_{t}{\hat{x}}_{t-1}^{-}$$

(8) $$P_{t}^{-}=F_{t}P_{t-1}F_{t}^{T}+Q_{t}$$

where ${\hat{x}}_{t}^{-}$ is the a priori state estimate at time t; $F_{t}$ is the state transfer matrix, initialised as a unit matrix, representing the assumption that the system remains stationary or moves at a constant velocity during each time step; $P_{t}^{-}$ is the prediction covariance matrix; $Q_{t}$ is the process noise covariance matrix representing the model’s uncertainty; and $P_{t-1}$ is the a posteriori state covariance matrix at the previous time step.

New observations are used to update the state estimates. In this step, the state estimates and covariance matrix are updated using the Kalman gain and measurement matrices of the Wi-Fi and camera:

(9) $$K_{t}=P_{t}^{-}H_{t}^{T}{\left(H_{t}P_{t}^{-}H_{t}^{T}+R_{t}\right)}^{-1}$$

(10) $${\hat{x}}_{t}={\hat{x}}_{t}^{-}+K_{t}\left(z_{t}-H_{t}{\hat{x}}_{t}^{-}\right)$$

(11) $$P_{t}=\left(I-K_{t}H_{t}\right)P_{t}^{-}$$

where $K_{t}$ is the Kalman gain; $H_{t}$ is the measurement matrix, initialized to a unit matrix, representing the assumption that these sensors directly observe each variable in the state space; $R_{t}$ is the measurement noise covariance matrix, which represents the uncertainty in the observations and is initialized to a small value to indicate the assumption that the noise level of these sensors is uniform and of medium size; $z_{t}$ is the actual measurement; and *I* is the identity matrix with the same dimensions as $P_{t}$. Algorithm 1 gives an update step for the FAKF, performing the standard Kalman filter update step to update the state estimates and covariance matrix by fusing observations from the camera and Wi-Fi.
**Algorithm 1** FAKF Update Step (Update)**Input**: Camera and WiFi measurements (z_camera, z_wifi) **Output**: Updated state estimate x and covariance matrix P 1: Initial parameters for the filter//Step 1. Camera Data Update2: $S_{camera}=H_{camera}PH_{camera}^{T}$ + $R_{camera}$;3: $K_{camera}={PH}_{camera}^{T}S_{camera}^{-1}$;4: ${innovation}_{camera}=Z_{camera}$ − $H_{camera}x$;5: x=x + $K_{camera}{innovation}_{camera}$;6: P = (I − $K_{camera}H_{camera})P$;// Step 2. WiFi Data Update7: $S_{wifi}=H_{wifi}PH_{wifi}^{T}+R_{wifi}$;8: $K_{wifi}=PH_{wifi}^{T}S_{wifi}^{-1}$;9: ${innovation}_{wifi}=Z_{wifi}$ − $H_{wifi}x$;10: x=x + $K_{wifi}{innovation}_{wifi}$;11: P = (I − $K_{wifi}H_{wifi})P$; // Step 3. Adaptive Sensor Fusion: Weighting the updates based on residuals12: ${residuals}_{combined}={weight}_{camera}{innovation}_{camera}$ + ${weight}_{wifi}{innovation}_{wifi}$;13: x=x + $K_{combined}{residuals}_{combined}$;14: P = (I − $K_{combined}H_{combined})P$;

In addition, an adaptive process is introduced to uniformly distribute the number of basic AKFs at the initial time. Algorithm 2 describes the adaptive tuning method of the FAKF, dynamically adjusting the noise parameters (both process and measurement noise) in the Kalman filter based on the data residuals from the camera and Wi-Fi, as well as adjusting the Kalman gain based on the weighted residuals.

In this process, outlier detection and weight adjustment are achieved by calculating the residuals between the predicted state and the actual measurements:

(12) $$e_{wifi}=z_{wifi}-H_{wifi}{\hat{x}}_{t}^{-}$$

(13) $$e_{camera}=z_{camera}-H_{camera}{\hat{x}}_{t}^{-}$$

The Euclidean modulus length of the residual vector is then calculated to quantify the magnitude of the residuals:

(14) $$||e_{wifi}||=\sqrt{e_{wifi}^{T}\cdot e_{wifi}}$$

(15) $$||e_{camera}||=\sqrt{e_{camera}^{T}\cdot e_{camera}}$$

**Algorithm 2** FAKF Adaptive Adjustment (Adapt)**Input**: Residuals from camera and WiFi data (innovation_camera, innovation_wifi) **Output**: Updated process and measurement noise covariance matrices 1: ${innovation}_{camera}=Z_{camera}$ − $H_{camera}x$;2: ${innovation}_{wifi}=Z_{wifi}$ − $H_{wifi}x$; // Step 1. Compute the weighted residuals for better decision making3: ${weight}_{camera}=exp($−0.5${innovation}_{camera}^{2}/R_{camera}$);4: ${weight}_{wifi}=exp($−0.5${innovation}_{wifi}^{2}/R_{wifi}$);5: beta = normalize(${weight}_{camera}$ + ${weight}_{wifi}$); // Step 2. Dynamically adjust noise parameters based on the residuals’ magnitude6: **if** max(innovation_camera, innovation_wifi) > observability threshold **then**7:       Q = 1.1Q;8:       $R_{camera}$= 0.9$R_{camera}$;9:       $R_{wifi}$= 0.9$R_{wifi}$;10: **else**        //Smooth noise adjustment based on sensor performance11:       Q = 0.98Q;12:       $R_{camera}$= 0.98$R_{camera}$;13:       $R_{wifi}$= 0.98$R_{wifi}$;14: **end if** // Step 3. Adjust the Kalman gain dynamically based on the weighted residuals15: $K_{camera\_adjusted}=K_{camera}{weight}_{camera}$;16: $K_{wifi\_adjusted}=K_{wifi}{weight}_{wifi}$;  // Step 4. Update state estimates based on weighted Kalman gains17: x=x + $K_{camera\_adjusted}{innovation}_{camera}+K_{wifi\_adjusted}{innovation}_{wifi}$;18: P = (I − $\;K_{camera\_adjusted}H_{camera}$ − $\;K_{wifi\_adjusted}H_{wifi})P$;

In order to detect whether the residuals are out of the normal range and thus identify outliers or model mismatches, an observability threshold, observability, is set with a value of 0.1. This threshold is used to control the sensitivity of outlier detection. An outlier is indicated when $||e_{wifi}||>observability\;or\;||e_{camera}||>observability$. At this point, the process noise covariance Q needs to be adjusted to increase model fitness and the measurement noise covariance R needs to be reduced to enhance confidence in the observations, thus improving the robustness and stability of the filter.

(16) $$Q\leftarrow a_{1}\cdot Q$$

(17) $$R\leftarrow \;a_{2}\cdot R$$

In this study, the process noise tuning factor $a_{1}$ was set to 1.1 and the measurement noise tuning factor $a_{2}$ was set to 0.9, choices based on the tuning principle of the Kalman filter as well as the system’s need to respond to dynamic changes. Specifically, increasing the process noise helps to improve the responsiveness of the filter to dynamic changes in the system. In practice, the system state may be affected by external perturbations or internal uncertainties, so moderately increasing the process noise can enhance the sensitivity of the filter to system state changes, avoid over-reliance on measurement data for system state estimation, and thus enhance the robustness of the system. On the other hand, reducing measurement noise helps to reduce the interference of unstable or occasionally distorted sensor data on state estimation. In the case of possible short-term fluctuations or errors in the sensors, reducing the measurement noise enables the filter to rely more on the process model of the system, which increases trust in high-quality sensor data and thus maintains a high estimation accuracy in the short term.

When residuals are within the normal range, the residuals’ magnitude updates the sensor data weights, reflecting each sensor’s contribution to the state estimation. If the innovation value exceeds a certain threshold, the weights decrease, indicating reduced trust in the measurements:

(18) $$w_{wifi}=\exp\left(-\frac{1}{2}\times {\left||e_{wifi}|\right|}^{2}\right)$$

(19) $$w_{camera}=\exp\left(-\frac{1}{2}\times {\left||e_{camera}|\right|}^{2}\right)$$

Weights are normalized to ensure they sum to one:

(20) $$\beta_{wifi}=\frac{w_{wifi}}{\left|\left|w_{wifi}+w_{camera}\right|\right|}$$

(21) $$\beta_{camera}=\frac{w_{camera}}{\left|\left|w_{wifi}+w_{camera}\right|\right|}$$

In order to improve the robustness of the perceptual capability, an improved AKF observability method is also proposed to measure the weight factor of the filter, as shown in Equation (22):

(22) $$O\left(t_{0},t_{f}\right)=\sum_{t=t_{0}}^{t_{f}}Tr\left({T_{t_{0}}}^{T}\cdot \left({H_{wifi}}^{T}H_{wifi}+{H_{c}}^{T}H_{c}\right)\cdot T_{t_{0}}\right)$$

where $T_{t_{0}}={F_{t}}^{t-t_{0}}$ is the power of the state transfer matrix $F_{t}$ at time step $t-t_{0}$ and Tr denotes the trace of the matrix, the sum of diagonal elements of the matrix.

Through this adaptive tuning mechanism, the filter dynamically adjusts its parameters based on real-time data, achieving optimal filtering performance under various environmental conditions. This approach is particularly suitable for scenarios with uncertainty or dynamic changes in sensor data, significantly improving the accuracy and robustness of positioning systems.

## 5. Experimental Setup and Experimental Results

### 5.1. Experimental Setting and Data Collection

#### 5.1.1. Hardware Parameters

Router: frequency (2.4 GHz), maximum transmit power of 20 dBm (100 mW), built-in antenna gain between 2 and 5 dBi.

USB camera: model DS-E14a with 1080p resolution, 30 frames per second frame rate, and 90-degree field of view.

Data processing device: Lenovo Xiaoxin 13Pro laptop with 13th generation Intel Core i7 processor, 16 GB LPDDR5 RAM and 1 TB NVMe SSD storage. This laptop is manufactured by Lenovo, headquartered in Beijing, China.

Mobile device for Wi-Fi signal acquisition: OPPO Reno 3; processor: MediaTek Helio P90; memory: 8 GB RAM; storage: 128 GB internal storage. The device is manufactured by OPPO, based in Dongguan, China.

#### 5.1.2. Description of the Experimental Environment

**Figure 3:**
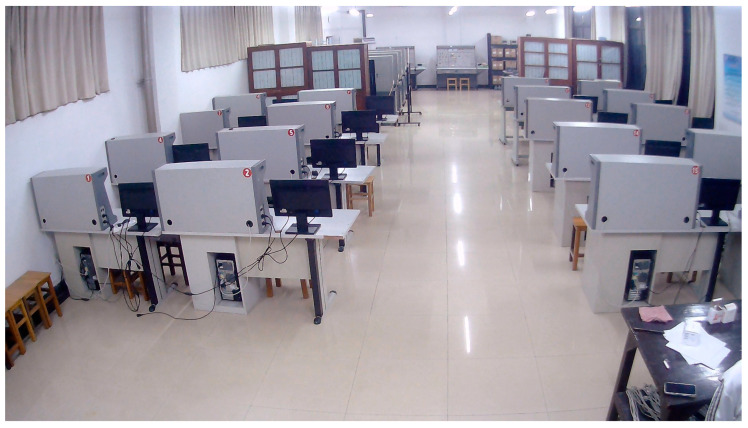
Real view of the experimental environment.

**Figure 4:**
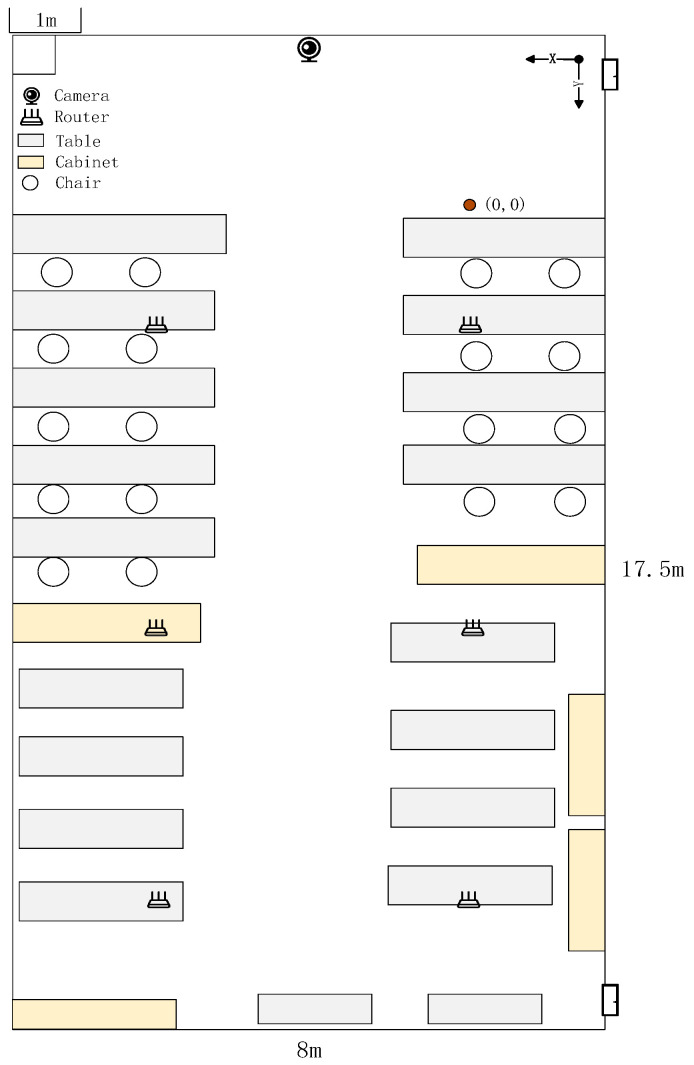
Top view plan of the experimental environment.

The experiment was conducted in a complex indoor laboratory setting, as depicted in Figure 3 and Figure 4. The laboratory measures 17.5 m in length, 8 m in width, and 3.5 m in height, featuring numerous embedded lab tables and large cabinets. This complexity poses challenges for pedestrian localization due to potential Wi-Fi signal interference and visual obstructions. For the experiments, six routers and one USB camera were strategically positioned to optimize coverage and minimize blind spots in the observed area.

#### 5.1.3. Establishment of the Wi-Fi Fingerprint Database

For Wi-Fi signal acquisition, Android software (version 1.0) written in Android Studio was used. The software captures the Received Signal Strength Indication (RSSI) of Wi-Fi signals, allowing for quantitative measurements of signal strength at specific locations. A comprehensive database of Wi-Fi signal fingerprints was created through extensive data collection, with no fewer than 3000 data collected at each collection point to ensure that it was not affected by environmental changes.

#### 5.1.4. Acquisition of Visual Information

A video camera connected to a laptop computer processing the input data was placed in the laboratory to facilitate visual data collection. The camera continuously monitored the area of interest to provide pedestrian movement data by capturing video frames. Using established external and internal calibration parameters (including focal length, lens distortion, and scene rotation), the system was able to accurately convert pixel coordinates into world coordinates to obtain precise pedestrian positions.

### 5.2. Comparison and Analysis of Experimental Results

#### 5.2.1. Experimental Methods

This study evaluated the performance of various positioning methods on different experimental paths. The experimenter walked in the test environment with a mobile phone, which performed Wi-Fi positioning by continuously scanning the surrounding Wi-Fi signals while capturing the experimenter’s visual data with a camera for frame-by-frame visual positioning analysis. The results of Wi-Fi positioning and visual positioning were then integrated by the proposed FAKF algorithm to achieve fused indoor positioning.

#### 5.2.2. Visual Positioning Component Evaluation

In order to demonstrate the effectiveness of the proposed method, this experiment included a comprehensive evaluation of the vision components. The evaluation metrics included precision, recall, and F1 score. The evaluation results are shown in Table 1.

The analysis of the results of the evaluation of the visual positioning component under different thresholds (Th) led to the following conclusions.

1. Accuracy: The accuracy of the model was 1 for all path and threshold conditions, indicating that it demonstrated an extremely good ability to correctly classify positive class samples without false positives. This result indicates that the model is very reliable in the prediction of positive class samples and is able to ensure that all positive class instances are identified without error.
2. Recall: Path 1 had a low recall at lower thresholds, but the recall gradually improved as the threshold increased and significantly improved at higher thresholds. In contrast, path 2 and path 3 showed better recall at all thresholds, especially at larger thresholds (Th = 2.70 m), where path 2 reached 0.880 and path 3 reached 0.846. This suggests that path 2 and path 3 were able to identify the positive class instances more efficiently, and especially at larger thresholds.
3. F1 score: The F1 scores of path 2 and path 3 gradually increased with the increase in the threshold, and they reached their best values of 0.936 and 0.917, respectively, at the threshold of 2.70 m, which indicates that these two paths achieved a better balance between precision and recall and that they can provide ideal comprehensive performance, especially under larger threshold conditions.

#### 5.2.3. Wi-Fi and Visual Fusion Positioning Evaluation

This section evaluates the experimental results of Wi-Fi and visual fusion positioning and compares the performance of the traditional Kalman-filter-based approach with the FAKF algorithm proposed in this paper. For performance comparison, the basic Kalman filter (KF) algorithm proposed by Kalman is used as a benchmark. To assess positioning accuracy, the positioning results of the three test paths are analyzed in this paper using root mean square error (*RMSE*) and mean absolute error (*MAE*).

(23) $$RMSE={\left(\frac{1}{N}\sum_{i=1}^{N}\left[{\left(x_{i}-x_{i}^{\prime }\right)}^{2}+{\left(y_{i}-y_{i}^{\prime }\right)}^{2}\right]\right)}^{1/2}$$

(24) $$MAE=\left(\frac{1}{N}\sum_{i=1}^{N}\left[\left|x_{i}-x_{i}^{\prime }\right|+\left|y_{i}-y_{i}^{\prime }\right|\right]\right)$$

where ($x_{i},y_{i}$) and ($x_{i}^{\prime },y_{i}^{\prime }$) are the estimated and actual position of the ith measurement point, respectively. Below are the trajectory diagrams of the three test paths and a summary table of the corresponding error metrics.

**Figure 5:**
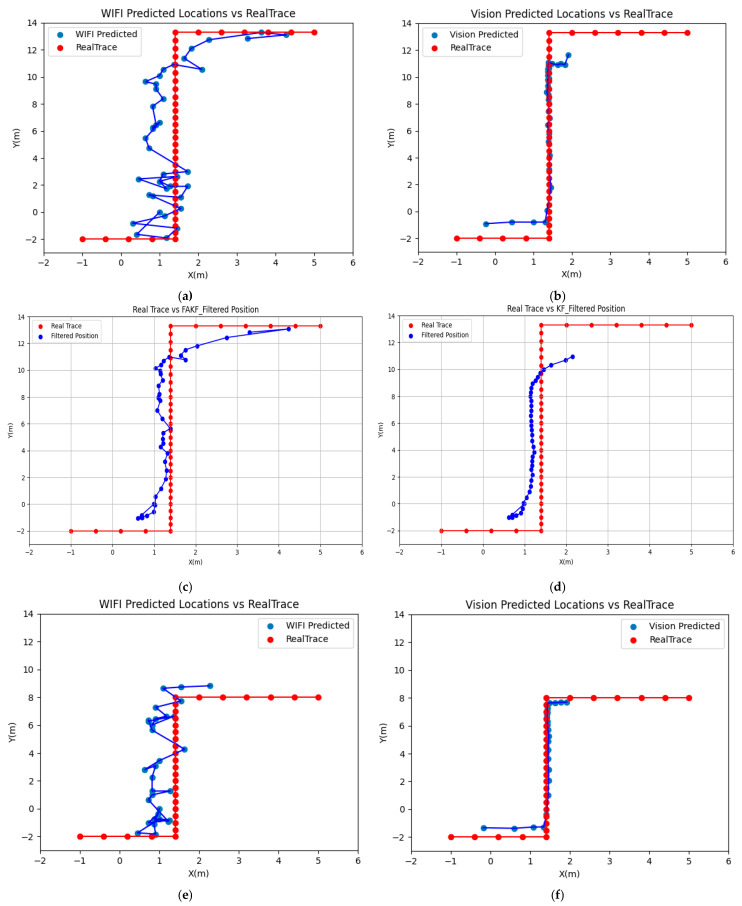

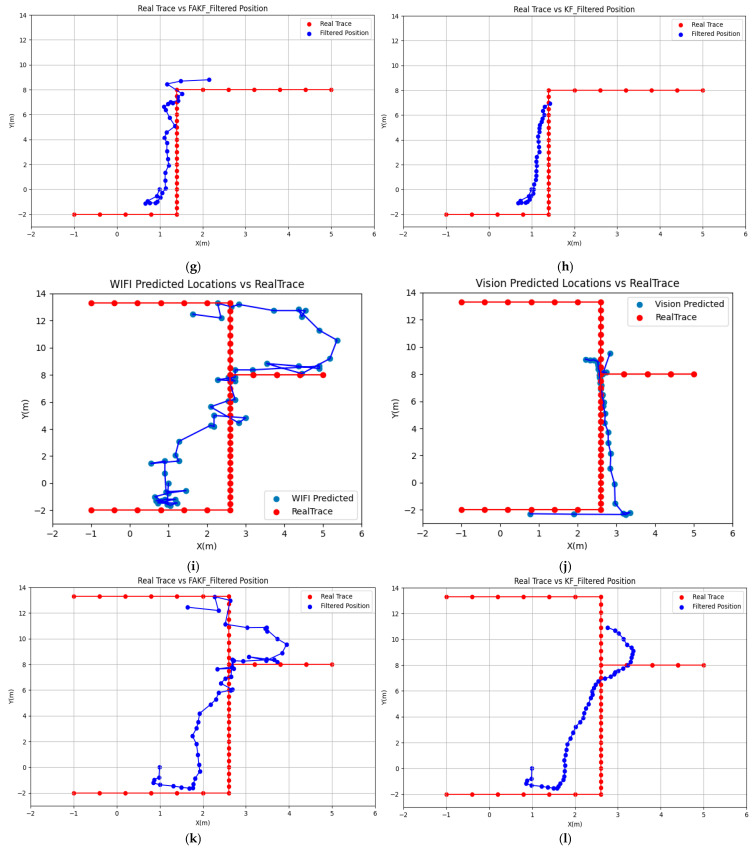
Trajectory maps of different positioning methods for three paths. (**a**) Wi-Fi positioning trajectory map of path 1; (**b**) visual positioning trajectory map of path 1; (**c**) FAKF positioning trajectory map of path 1; (**d**) KF positioning trajectory map of path 1; (**e**) Wi-Fi positioning trajectory map of path 2; (**f**) visual positioning trajectory map of path 2; (**g**) FAKF positioning trajectory map of path 2; (**h**) KF positioning trajectory map of path 2; (**i**) Wi-Fi positioning trajectory map of path 3; (**j**) visual positioning trajectory map of path 3; (**k**) FAKF positioning trajectory map of path 3; (**l**) KF positioning trajectory map of path 3.

The experimental results show that the fusion positioning method using the FAKF algorithm exhibited excellent performance on different paths, with significantly lower positioning errors compared with the single-sensor data. As shown in Figure 5a–d, the Wi-Fi positioning trajectory in path 1 was rougher, and the visual positioning trajectory showed a large deviation. In contrast, the FAKF positioning trajectory was smoother and closely followed the actual path. As can be seen from Table 2, the RMSE for Wi-Fi positioning was 1.309 m, and the RMSE for visual positioning was 1.793 m. In contrast, the RMSE of the FAKF was reduced to 0.885 m and the MAE was only 0.683 m, which indicates a significant improvement in positioning accuracy with fusion positioning. When using the KF fusion method, the RMSE increased to 1.585 m and the MAE increased to 1.121 m. This suggests that KF could provide some degree of smoothing, but its performance was not comparable to that of the FAKF algorithm and it failed to integrate the complementary information from the Wi-Fi and the visual data as efficiently as the FAKF algorithm.

For path 2 (Figure 5e–h), although the visual positioning results were improved due to the smaller walking range, their positioning results were still unsatisfactory. Specifically, the RMSE of Wi-Fi positioning was 1.464 m and that of visual positioning was 1.050 m. After applying the FAKF algorithm, the trajectory was significantly improved; the RMSE of the FAKF was reduced to 0.897 m, and the MAE was only 0.591 m, which indicates that the fusion positioning method was effective. The performance improvement of the KF fusion method was more limited compared with the FAKF algorithm, with an RMSE of 1.442 m and an MAE of 1.143 m. Although Kalman filtering helped to reduce the error to a certain extent, it was not as effective as the FAKF algorithm in dealing with the fusion of Wi-Fi and visual data.

In path 3 (Figure 5i–l), both Wi-Fi positioning and visual positioning showed different degrees of deviation because the walking range became larger and the walking path became more complex. According to the data, the RMSE of Wi-Fi positioning was 1.439 m and the RMSE of visual positioning was 1.199 m. In comparison, the RMSE for the FAKF was 1.060 m and the MAE was 0.835 m. The error of FAKF positioning increased due to the change in path, but it still showed a significant improvement in positioning accuracy compared with the single sensor. When the path complexity increased, the RMSE of the KF-based fusion positioning method was 1.842 m and the MAE was 1.538 m. In contrast, the FAKF algorithm performed better in this scenario, probably due to the fact that the FAKF has a higher processing efficiency in dealing with nonlinear properties and high noise levels in data.

**Figure 6:**
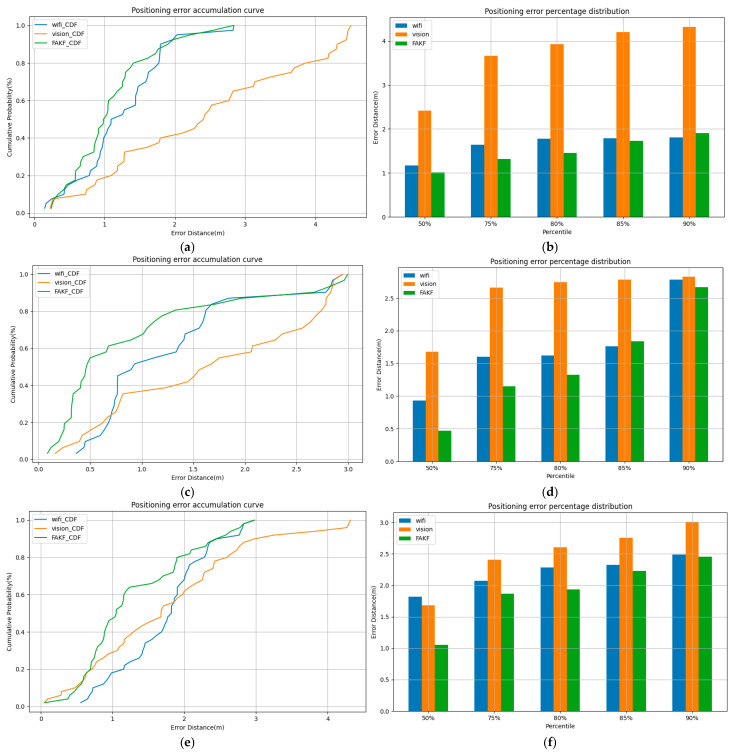
Positioning CDF plots for different positioning methods for three paths. (**a**) CDF plot for path 1; (**b**) different percentile errors for path 1; (**c**) CDF plot for path 2; (**d**) different percentile errors for path 2; (**e**) CDF plot for path 3; (**f**) path 3 with different percentile errors.

In complex indoor environments, this FAKF method reduces the uncertainty associated with a single technique by combining data from Wi-Fi positioning and visual positioning, effectively improving the robustness and accuracy of positioning. These experimental results validate the practical application potential of the method and highlight its broad application prospects. This study also examined the positioning error rates of different methods across various paths, as depicted in Figure 6. The percentile error data for the three paths are detailed in Table 3, Table 4 and Table 5.

In path 1, as shown in Table 3, the median error for Wi-Fi positioning was 1.17 m and the 90% cumulative distribution function (CDF) error was 1.81 m. The median error of visual positioning was 2.42 m and the 90% CDF error was 4.32 m, showing a large instability. In contrast, the median error of fused positioning was 1.01 m and the 90% CDF error was 1.91 m, which were smaller errors compared with the median error of Wi-Fi positioning and a smaller increase compared with visual positioning, which shows that fused positioning is still effective in most cases.

In path 2, as shown in Table 4, the Wi-Fi positioning error increased from a median error of 0.93 m to a 90% CDF error of 2.78 m, which was a significant increase in error. The visual positioning error increased from a median error of 1.68 m to a 90% CDF error of 2.83 m, with a large median error. In contrast, the median error of fused positioning was only 0.47 m, and its 90% CDF error of 2.67 m was lower than the 90% error of the single technique, suggesting that the fused positioning technique provides more accurate results in most cases.

In path 3, as shown in Table 5, there was a large error fluctuation from the median error of 1.78 m for Wi-Fi positioning and 2.93 m for the 90% CDF error. Visual positioning increased from a median error of 1.68 m to 3.01 m for the 90% CDF error, with a similar range of fluctuation as Wi-Fi positioning. Fusion positioning had a median error of 1.05 m and a 90% CDF error of 2.46 m, both of which were lower than the single-technology errors and showed better overall stability.

The above experimental results show that the fused positioning method was overall better than single Wi-Fi or visual positioning techniques, confirming the effectiveness of this fused positioning technique in reducing errors and improving accuracy in complex indoor environments.

## 6. Conclusions

With the rapid development of smart devices and IoT technologies, there is a growing demand for accurate indoor positioning. However, complex indoor environments pose a great challenge to single-sensor positioning methods. To address this problem, this study proposes an indoor fusion positioning method that combines Wi-Fi and vision data as a way to improve the accuracy and reliability of positioning systems. This technique improves the accuracy and robustness of positioning by integrating data from cameras and Wi-Fi sensors and dynamically adjusting the noise covariance using adaptive Kalman filtering so that it can effectively cope with complex and changing indoor environments.

Experiments showed that the fusion positioning method performed well in improving the accuracy of positioning, and its root mean square error was significantly lower than that of single Wi-Fi positioning or visual positioning. Comparing the real trajectories with the fused positioning results further validated the effectiveness and practicality of the method. Therefore, the adaptive Kalman filtering method combining Wi-Fi data and visual data proposed in this study provides a reliable and efficient solution for indoor positioning in complex environments.

However, the proposed FAKF fusion method still has some limitations in indoor positioning. The effectiveness of the method is highly dependent on the specific characteristics of the indoor environment, such as Wi-Fi signal coverage, camera field of view, and lighting conditions. Environmental factors (e.g., walls, furniture layout, and signal interference) may affect the positioning accuracy, limiting the applicability of the method in some specific environments. Although the FAKF algorithm is able to cope with environmental noise and uncertainty to a certain extent, its positioning performance may still be significantly affected in extreme environments (e.g., areas with severe signal attenuation or high interference).

To address these limitations, future research can optimize and improve them in the following directions:

1. Optimization of visual positioning methods: The performance of the visual positioning methods used in this study in large-scale environments has the potential to be further improved. Future work should focus on optimizing the visual positioning techniques to improve their accuracy in complex scenes, especially in poor visible light conditions or with large scene variations, so as to enhance the accuracy of the overall data fusion positioning results.
2. Upgrading of fused positioning methods: The FAKF used in this study relies on an adaptive adjustment mechanism for data fusion. Future research can further explore and implement more advanced sensor fusion strategies, such as a multi-sensor data fusion framework, while introducing more sophisticated fusion techniques to more effectively cope with nonlinear problems and variable factors in complex environments.

## Figures and Tables

**Table 1:** Visual positioning component evaluation results.

Path	Metrics	Th = 1.60 m	Th = 1.80 m	Th = 2.00 m	Th = 2.50 m	Th = 2.70 m
Path 1	precision	1.0000	1.0000	1.0000	1.0000	1.0000
	recall	0.3056	0.3611	0.3611	0.5278	0.5556
	F1 score	0.4681	0.5306	0.5306	0.6909	0.7143
Path 2	precision	1.0000	1.0000	1.0000	1.0000	1.0000
	recall	0.5600	0.6000	0.6000	0.7600	0.8800
	F1 score	0.7179	0.7500	0.7500	0.8636	0.9362
Path 3	precision	1.0000	1.0000	1.0000	1.0000	1.0000
	recall	0.5128	0.5897	0.6410	0.7949	0.8462
	F1 score	0.6780	0.7419	0.7813	0.8857	0.9167

**Table 2:** Positioning error results table.

Path	Wi-Fi RMSE (m)	Visual RMSE (m)	FAKF RMSE (m)	FAKF MAE (m)	KF RMSE (m)	KF MAE (m)
Path 1	1.30900	1.79313	0.88482	0.68337	1.58489	1.12138
Path 2	1.46400	1.04980	0.89663	0.59074	1.44230	1.14330
Path 3	1.43900	1.19865	1.05999	0.83463	1.84172	1.53824

**Table 3:** Percentile error data table for path 1.

	50%	75%	80%	90%
Wi-Fi positioning error (m)	1.17	1.64	1.77	1.81
Visual positioning error (m)	2.42	3.67	3.93	4.32
FAKF positioning error (m)	1.01	1.31	1.45	1.91

**Table 4:** Percentile error data table for path 2.

	50%	75%	80%	90%
Wi-Fi positioning error (m)	0.93	1.60	1.62	2.78
Visual positioning error (m)	1.68	2.66	2.74	2.83
FAKF positioning error (m)	0.47	1.15	1.32	2.67

**Table 5:** Percentile error data table for path 3.

	50%	75%	80%	90%
Wi-Fi positioning error (m)	1.78	2.52	2.66	2.93
Visual positioning error (m)	1.68	2.41	2.61	3.01
FAKF positioning error (m)	1.05	1.87	1.93	2.46

## Data Availability

No new data were created or analyzed in this study. Data sharing is not applicable to this article.
